# Maternal Obesity Alters Placental and Umbilical Cord Plasma Oxidative Stress, a Cross-Sectional Study

**DOI:** 10.3390/ijms251910866

**Published:** 2024-10-09

**Authors:** Thanyawan Jantape, Kiattisak Kongwattanakul, Silvia M. Arribas, Pilar Rodríguez-Rodríguez, Metee Iampanichakul, Wannapa Settheetham-Ishida, Sophida Phuthong

**Affiliations:** 1 Department of Physiology, Faculty of Medicine, Khon Kaen University, Khon Kaen 40002, Thailand; thanyawan.j@kkumail.com (T.J.); meteiam@kku.ac.th (M.I.); wannapa@kku.ac.th (W.S.-I.); 2 Department of Obstetrics and Gynecology, Faculty of Medicine, Khon Kaen University, Khon Kaen 40002, Thailand; kiattisak@kku.ac.th; 3 Department of Physiology, Faculty of Medicine, Universidad Autónoma de Madrid, 28029 Madrid, Spain; silvia.arribas@uam.es (S.M.A.); pilar.rodriguezr@uam.es (P.R.-R.); 4 Human High Performance and Health Promotion Research Institute, Khon Kaen University, Khon Kaen 40002, Thailand

**Keywords:** maternal obesity, placenta, oxidative stress, antioxidants

## Abstract

Maternal obesity has been shown to impair the oxidative status in the placenta and newborns, potentially leading to adverse pregnancy outcomes and long-term effects on the programming of offspring metabolic status. This study aimed to investigate the impact of maternal obesity on maternal and umbilical cord plasma oxidative status, as well as placental oxidative adaptation. Maternal obesity (*n* = 20), defined as a pre-pregnancy BMI ≥ 25 kg/m^2^, and maternal leanness (*n* = 20), defined as a pre-pregnancy BMI < 23 kg/m^2^, were the group categories used in this study. Both groups were matched according to gestational age at delivery. Maternal blood, umbilical cord blood, and placental tissue were collected to assess nutritional content (cholesterol, triglyceride, and protein), oxidative stress markers (MDA and protein carbonyl), and antioxidant activity (SOD and catalase). Placental protein expression (SOD2, catalase, UCP2, and Nrf2) was evaluated using Western blot analysis. Catalase activity in maternal plasma significantly increased in the maternal obesity group (*p* = 0.0200), with a trend toward increased MDA and protein carbonyl levels. In umbilical cord plasma, triglyceride, protein carbonyl, and catalase activity were significantly elevated in the maternal obesity group compared with the lean controls (*p* = 0.0482, 0.0291, and 0.0347, respectively). Placental protein expression analysis revealed significantly decreased SOD2 (*p* = 0.0011) and catalase (*p* < 0.0001), along with Nrf2 downregulation (*p* < 0.0001). An increase in mitochondrial antioxidant UCP2 expression was observed (*p* = 0.0117). The neonatal protein carbonyl levels positively correlated with placental protein carbonyl (r = 0.7405, *p* < 0.0001) and negatively correlated with maternal catalase activity (r = −0.4332, *p* = 0.0052). This study thus provides evidence that maternal obesity is associated with placental and fetal oxidative stress, alongside a concurrent increase in placental antioxidant UCP2 expression.

## 1. Introduction

Obesity is one of the most significant global public health challenges of the 21st century, with a rising prevalence among women of reproductive age [1]. The criteria for obesity in Asians define the condition as excessive fat accumulation, with a body mass index (BMI) ≥ 25 kg/m^2^ [2,3]. This condition is particularly concerning in women of reproductive age due to its impact on pregnancy, affecting the health of at least two generations [4]. Maternal obesity represents a stress condition that can lead to impaired feto-placental development and function, potentially exerting long-term negative effects on metabolic programming in the offspring [5,6,7].

During pregnancy, metabolic changes occur in women to ensure sufficient nutrient availability for the developing placenta and fetus [8]. Maternal obesity has been linked to metabolic dysregulation, which disrupts both maternal and fetal metabolic and oxidative status [9,10]. The placenta, as the key organ facilitating maternal–fetal interaction, regulates critical aspects of the intrauterine environment, including oxygen and nutrient delivery, waste removal, and neutralization of reactive oxygen species (ROS) [11]. Thus, oxidative adaptation may modulate the effects of maternal obesity on fetal oxidative stress [12,13].

Pregnancy is considered a state of maternal oxidative stress that can affect the placenta. However, this oxidative stress is exacerbated in obese pregnant women [11,12,13]. Our previous study on placental adaptation to maternal malnutrition in a rat model demonstrated an oxidative imbalance in the placenta [14]. Various enzymes contribute to placental antioxidant defenses, including superoxide dismutase (SOD), catalase, and glutathione peroxidase (GPx) [15]. Additionally, due to the mitochondria-rich nature of the placental tissue, mitochondrial antioxidant uncoupling protein 2 (UCP2) has been proposed as a regulator of oxidative stress defense in the placenta [15,16]. Notably, UCP2 expression has been found to be responsive to changes in maternal nutrition [17]. A recent study reported a negative correlation between maternal BMI and placental SOD expression [18]. Furthermore, maternal oxidative stress markers have been positively correlated with neonatal oxidative stress markers [19]. However, findings on the placental antioxidant response in obese pregnancies are inconsistent. Most studies have been conducted in non-Asian populations using different BMI cut-off points [12,20]. Some studies have reported increased placental antioxidant levels, such as SOD, catalase, and GSH, alongside reduced maternal plasma antioxidant activity [12,20]. In contrast, another study found reduced total antioxidant capacity in the placenta, along with decreased SOD and catalase activity and increased oxidative and nitrosative stress markers [21]. Taken together, these findings suggest that maternal obesity may contribute to a placental oxidative imbalance, potentially predisposing the fetus to oxidative stress.

The aim of this study was to investigate the effect of maternal obesity during pregnancy on maternal and umbilical cord plasma oxidative status, as well as placental oxidative adaptation. We hypothesized that compromised placental antioxidant defenses may contribute to neonatal oxidative stress under adverse maternal conditions.

## 2. Results

Demographic and pregnancy-outcome data are presented in Table 1. There were no significant differences in maternal age or gestational age at delivery between the maternal lean and the maternal obesity groups (*p* > 0.05). However, pre-pregnancy weight, pre-pregnancy BMI, and weight at delivery were significantly higher in the maternal obesity group compared to the normal-weight group (*p* < 0.0001). The percentage of male neonates did not differ significantly between obese and normal-weight women (*p* > 0.05). Neonatal birth weight and length also did not differ significantly between the two groups (*p* > 0.05). Interestingly, the placental weight of the maternal obesity group was significantly higher compared to that of the maternal lean group (*p* = 0.0224), and the feto-placental ratio was significantly lower in the maternal obesity group (*p* = 0.0374).

**Figure 1:**
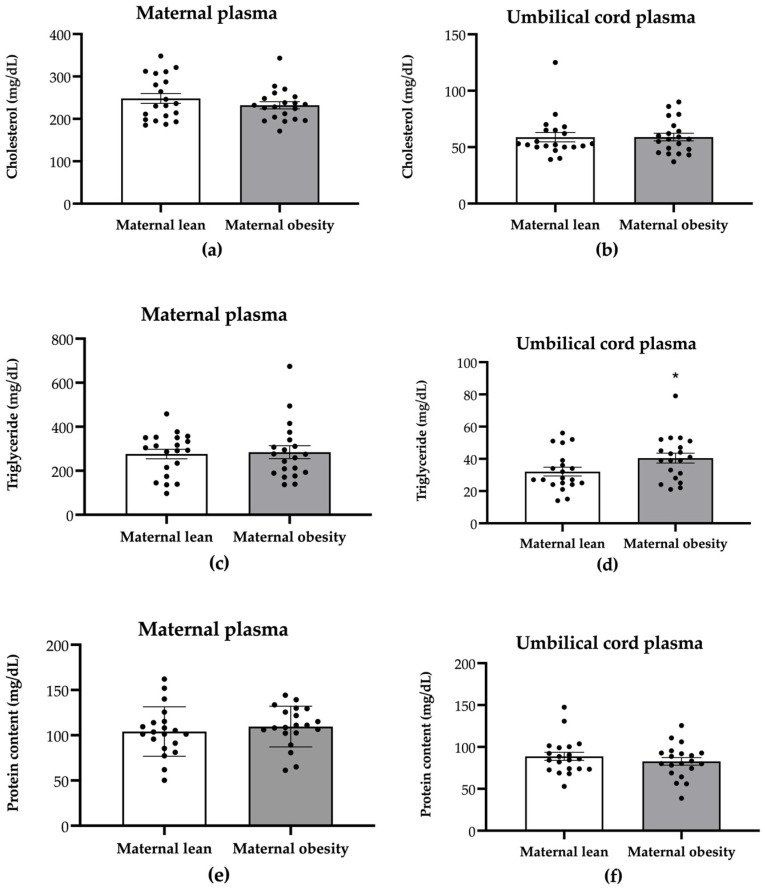
Maternal plasma and umbilical cord plasma levels of total cholesterol (**a**,**b**), triglyceride (**c**,**d**), and protein (**e**,**f**) in maternal lean and maternal obesity groups. Results are expressed as mean ± SEM, *n* = 20 each. *p*-value was tested using Student’s *t*-test; * *p* < 0.05 relative to the maternal lean group.

To assess the impact of maternal obesity on the nutritional content on both the maternal and the neonatal sides, we assessed total cholesterol, triglyceride, and protein content in maternal and umbilical cord plasma. There were no significant differences in the maternal plasma levels of cholesterol, triglyceride, or protein between the maternal obesity group and the maternal lean control group (cholesterol: *p* = 0.3081; triglyceride: *p* = 0.8163; protein: *p* = 0.4884; Figure 1a,c,e, respectively). However, a significantly higher level of umbilical cord plasma triglyceride was observed in the maternal obesity group (*p* = 0.0482; Figure 1d) compared with the lean group, while no significant differences were found in umbilical cord plasma cholesterol or protein content (*p* = 0.9776 and *p* = 0.3900, respectively; Figure 1b,f).

**Figure 2:**
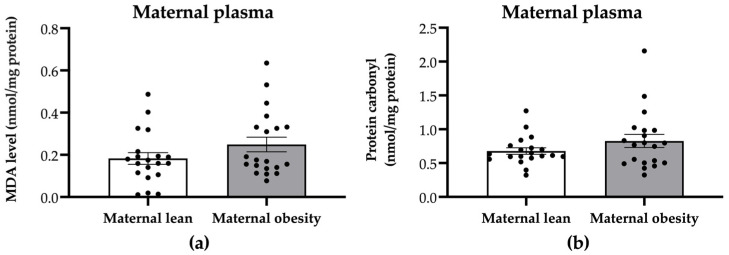
Levels of (**a**) malondialdehyde (MDA) and (**b**) protein carbonyl in the maternal plasma of the maternal lean and maternal obesity groups. Results are expressed as mean ± SEM; *n* = 20 each. *p*-value was tested using Student’s *t*-test.

To evaluate whether maternal plasma oxidative stress markers were affected by maternal obesity, we examined the levels of MDA and protein carbonyl. There were no significant differences in the maternal plasma MDA or protein carbonyl levels between the maternal obesity group and the maternal lean control group (*p* = 0.1452 and *p* = 0.1744, respectively; Figure 2a,b).

**Figure 3:**
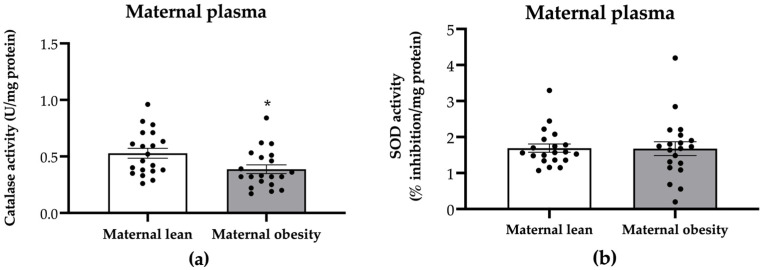
Activity of (**a**) catalase and (**b**) superoxide dismutase (SOD) in the maternal plasma of the maternal lean and maternal obesity groups. Results are expressed as mean ± SEM; *n* = 20 each; *p*-value was tested using Student’s *t*-test; * *p* < 0.05 relative to the maternal lean group.

Maternal plasma catalase activity was significantly decreased in the maternal obesity group compared to the maternal lean group (*p* = 0.0201; Figure 3a), while SOD activity showed no significant difference between the groups (*p* = 0.9526; Figure 3b).

**Figure 4:**
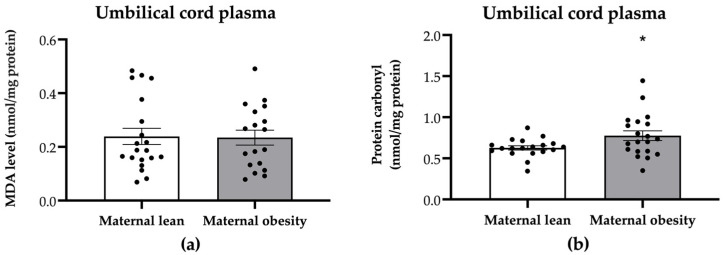
Levels of (**a**) malondialdehyde (MDA) and (**b**) protein carbonyl in the umbilical cord plasma of the maternal lean and maternal obesity groups. Results are expressed as mean ± SEM; *n* = 20 each. *p*-value was tested using Student’s *t*-test. * *p* < 0.05 relative to the maternal lean group.

The oxidative stress markers in umbilical cord plasma are presented in Figure 4. The protein carbonyl levels were significantly higher in the umbilical cord plasma of the maternal obesity group compared to the maternal lean group (*p* = 0.0291; Figure 4b). However, there were no significant differences in umbilical cord plasma MDA levels between the maternal obesity group and the maternal lean control (*p* = 0.9162; Figure 4a).

**Figure 5:**
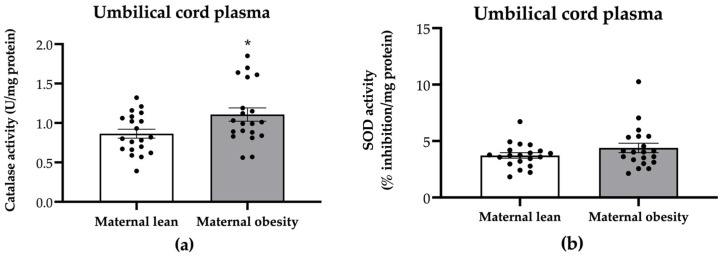
Activity of (**a**) catalase and (**b**) superoxide dismutase (SOD) in the umbilical cord plasma of the maternal lean and maternal obesity groups. Results are expressed as mean ± SEM; *n* = 20 each. *p*-value was tested using Student’s *t*-test; * *p* < 0.05 relative to the maternal lean group.

Regarding the antioxidant activity on the neonatal side, we assessed catalase and SOD activity in umbilical cord plasma. Notably, the umbilical cord plasma from the maternal obesity group exhibited significantly higher catalase activity (*p* = 0.0347; Figure 5a), while SOD activity showed no significant difference compared to that in the maternal lean group (*p* = 0.1739; Figure 5b).

**Figure 6:**
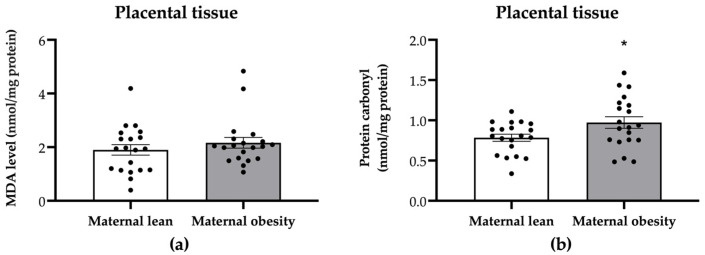
Level of (**a**) malondialdehyde (MDA) and (**b**) protein carbonyl in the placental tissue of the maternal lean and maternal obesity groups. Results are expressed as mean ± SEM; *n* = 20 each; *p*-value was tested using Student’s *t*-test. * *p* < 0.05 relative to the maternal lean group.

Regarding oxidative markers in the placental tissue, the MDA level was not significantly different between the maternal obesity group and the maternal lean control group (*p* = 0.3415; Figure 6a). However, the placental protein carbonyl level was significantly higher in the maternal obesity group compared to the maternal lean group (*p* = 0.0300; Figure 6b).

**Figure 7:**
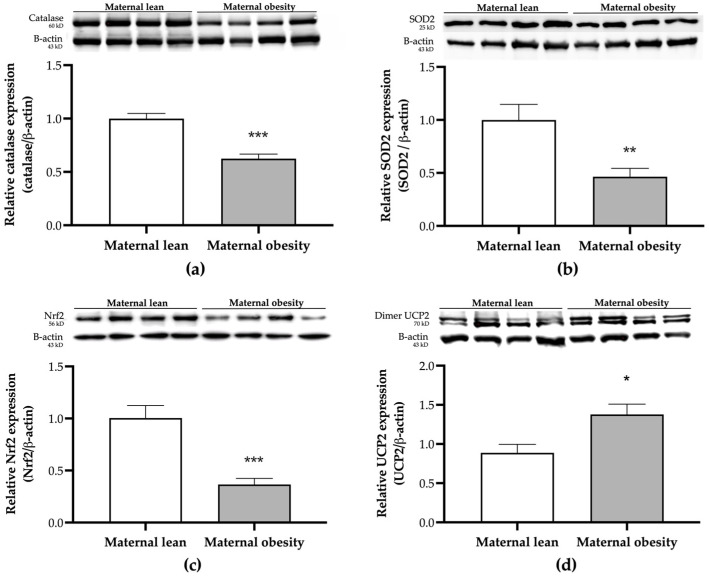
Placental protein expression of (**a**) catalase, (**b**) SOD2, (**c**) Nrf2, and (**d**) UCP2, measured using Western blot analysis in the maternal lean and maternal obesity groups (*n* = 12/group). Results are expressed as mean ± SEM; *n* = 12 each; *p*-value was tested using the Man–Whitney test; * *p* < 0.05, ** *p* < 0.01, *** *p* < 0.001 relative to the maternal lean group.

The relative placental antioxidant protein expressions are presented in Figure 7a–d. Catalase and SOD2 expressions in the placental tissue were significantly lower in the maternal obesity group compared to the maternal lean control (*p* < 0.0001 and *p* = 0.0011, respectively; Figure 7a,b). Additionally, the expression of the signaling pathway Nrf2 protein was significantly reduced in the maternal obesity group (*p* < 0.0001; Figure 7c). In contrast, the level of the mitochondrial antioxidant UCP2 was significantly higher in the maternal obesity group compared to the maternal lean group (*p* = 0.0104; Figure 7d).

Next, we explored the correlations between newborn oxidative levels and placental and maternal oxidative status. The level of umbilical cord protein carbonyl was positively correlated with that of placental protein carbonyl (r = 0.7405, *p* < 0.0001) and negatively correlated with maternal catalase activity (r = −0.4332, *p* = 0.0052). A trend of negative correlation was also observed between umbilical cord protein carbonyl and placental catalase expression (r = −0.3942; *p* = 0.0566). Additionally, umbilical cord protein carbonyl expression showed a positive correlation with maternal pre-pregnancy weight, pre-pregnancy BMI, and weight at delivery (r = 0.3621, 0.3832, and 0.3168, respectively; *p* = 0.0217, 0.0147, and 0.0464, respectively) (Table 2).

## 3. Discussion

In this study, maternal obesity maintained maternal plasma oxidative balance, as suggested by the trend of increased MDA and protein carbonyl levels alongside decreased plasma catalase activity. Interestingly, placental oxidative adaptation was observed, characterized by reduced placental catalase and SOD2 expression, coupled with enhanced UCP2 expression. This placental response was linked to the downregulation of the Nrf2 signaling pathway. Additionally, umbilical cord protein carbonyl and antioxidant catalase activity were significantly increased, indicating oxidative imbalance in the newborns. Neonatal oxidative stress positively correlated with placental oxidative stress and negatively correlated with maternal catalase activity.

Obesity during pregnancy has been associated with dysregulated lipid metabolism, which may expose the fetus to an adverse intrauterine environment [22,23,24]. This condition increases the risk of obstetric complications and adverse neonatal health outcomes at birth, while also affecting fetal metabolic programming and potentially influencing long-term health [25]. Maternal obesity has been linked to alterations in plasma lipid, glucose, and protein levels [26,27]. In our study, maternal and umbilical cord cholesterol and triglyceride levels in both groups fell within the normal ranges for the third trimester of pregnancy, indicating adequate nutritional health in both mothers and their newborns [28,29]. We did not observe any significant differences in total cholesterol, triglyceride, or protein levels in the maternal plasma of the obesity group. This contrasts with previous studies reporting elevated cholesterol and triglyceride levels in obese pregnancies. A possible explanation for this discrepancy could be differences in the study populations, which may result in varying genetic predispositions to dyslipidemia, as well as differences in BMI that might influence the severity of obesity [24,27,30]. However, the umbilical cord plasma from the maternal obesity group had higher triglyceride levels compared to the that from the lean control. Similar findings have been reported, showing elevated triglyceride levels in fetuses and placentas of mothers with metabolic disorders [11,20,31]. Additionally, there was evidence of dysregulation in placental fatty acid transporters and lipase enzymes in mothers with complications such as metabolic disorders, which directly affects lipid transfer from the placenta to the fetus [31,32,33]. We hypothesize that maternal obesity may lead to an increased transport of free fatty acids to the fetus, resulting in greater fat accumulation in the form of triglycerides. Elevated triglyceride levels can contribute to inflammation and potentially impact organ development and function [34,35]. Consequently, fetuses with elevated triglyceride levels are more likely to become obese in childhood and adulthood [33,36]. Therefore, our findings suggest that, even when the maternal nutrient levels are within normal ranges, maternal obesity can impair the fetal lipid metabolism, potentially increasing the risk of metabolic disorders later in life.

Additionally, maternal overnutrition has been correlated with an altered oxidative status in both the placenta and the newborn [7,35,37]. In normal pregnancy, increased ROS production is typically accompanied by an upregulation of antioxidant enzymes [38]. However, the oxidative status in maternal obesity remains controversial [19,21,39]. To investigate this, we assessed the oxidative status in maternal plasma by measuring lipoperoxidation products (MDA and protein carbonyl) alongside catalase and SOD activity. Our results showed no significant changes in the plasma MDA and protein carbonyl levels in the maternal obesity group, but there was a reduction in catalase activity. Catalase is a crucial antioxidant responsible for reducing free radicals and converting the final products of lipid peroxidation into inactive forms [40]. The reduced catalase activity observed in maternal obesity could stem from an impaired antioxidant response or from the depletion of antioxidants due to prolonged ROS exposure [20]. This impairment may be linked to diminished Nrf2 signaling or to the overactivation of xanthine oxidase, both of which have been associated with maternal metabolic disorders [41,42]. Our findings align with previous studies, which have shown that pregnant women with obesity exhibit elevated levels of plasma MDA and other oxidative stress markers, along with alterations in plasma antioxidants [19,20]. Hence, our data suggest that maternal obesity, even in the absence of obstetric complications, is associated with oxidative imbalance.

There is evidence that maternal nutrition influences the oxidative status in the placenta [18]. Maintaining a proper balance between pro-oxidants and antioxidants is essential for preserving placental homeostasis as part of its adaptive response [12]. Under physiological conditions, reactive molecules play a role in cell signaling, regulating normal placental development and functions such as angiogenesis, nutrient transport, hormone production, and oxidative control. However, excessive radical production can disrupt placental function [43]. To assess the impact of maternal obesity on placental oxidative alterations, we evaluated the oxidative status in the placenta. As expected, maternal obesity was associated with an altered placental oxidative status, demonstrated by a significant increase in placental protein carbonyl levels and a compromised antioxidant enzyme expression. Specifically, we observed decreased SOD2 and catalase levels, as well as reduced expression of the downstream signaling pathway Nrf2. In contrast, mitochondrial antioxidant UCP2 expression was elevated. SOD2 and catalase are general antioxidants located in mitochondria and peroxisomes, respectively [20], and their expression is regulated by Nrf2, a key transcriptional regulator of cellular responses to oxidative stress [44]. The reduction in placental SOD2 and catalase suggests the inactivation of the antioxidant system in the placenta of obese mothers. Furthermore, the significant reduction in Nrf2 protein expression observed in this study provides a clear mechanistic explanation for the impaired placental antioxidant defense. Several studies support our findings, showing elevated levels of oxidative stress markers along with downregulated SOD, catalase, and GPx in the placentas of mothers with metabolic disorders [12,18,20]. UCP2 is also recognized as a key mitochondrial antioxidant in the placenta [14]. Given the high mitochondrial activity in the placenta and the observed reductions in SOD and catalase expression, the upregulation of UCP2 may represent a compensatory response to the pro-oxidant state induced by maternal metabolic abnormalities. Our findings highlight the role of UCP2 in maintaining oxidative balance in the placenta, as previously reported [13,14]. However, despite the upregulation of UCP2, which may reflect placental adaptation to increased ROS production, there was a rise in placental protein carbonyl levels, indicating oxidative stress. While the mode of delivery has been linked to changes in placental oxidative stress [45], there was no significant difference in delivery mode between the two groups, ruling out its influence on the elevated placental protein carbonyl levels observed in maternal obesity. This suggests that the antioxidant response in the placentas of obese mothers may be insufficient to restore oxidative balance, potentially leading to placental dysfunction. Further studies on placental enzymatic activity could provide additional insights into this mechanism.

In addition to maternal and placental changes, we also observed alterations in the oxidative status of the newborn. We hypothesized that placental dysfunction induced by maternal obesity might impair the placenta’s ability to protect the fetus from adverse oxidative effects. Our results demonstrated significantly increased protein carbonyl levels and a non-significant trend toward higher MDA levels in the umbilical cord plasma from infants born to obese mothers compared to that from infants born to normal-weight mothers. Protein carbonylation, an irreversible oxidative modification, is a general marker of oxidative stress [46]. Elevated lipid peroxidation in the umbilical cord plasma further supports the presence of oxidative stress, as lipids are primary targets of radical reactions. The increased fatty acid transfer to the fetus through the placenta in maternal obesity may contribute to this oxidative stress [35]. Our findings are consistent with other studies reporting higher levels of oxidative stress markers in newborns of obese mothers [37,47]. Regarding the neonatal antioxidant response, our study found significantly increased umbilical cord plasma catalase activity without changes in SOD activity. Previous research showed that catalase plays a critical role in responding to oxidative stress under high H₂O₂ conditions, whereas SOD remains unaffected [48,49], which may explain the lack of alteration in SOD activity. The observed increase in catalase activity in newborns exposed to maternal obesity suggests an active antioxidant defense system, likely stimulated to neutralize the harmful effects of ROS. The simultaneous increase in protein oxidative products and antioxidant enzyme activity indicates that maternal obesity compromises neonatal oxidative status. To determine the connection between oxidative stress in newborns and placental oxidative status, we assessed the correlation between umbilical cord protein carbonyl and placental oxidative markers. The umbilical cord protein carbonyl levels were positively correlated with the placental protein carbonyl levels, while negatively correlated with placental catalase expression and maternal catalase activity. These findings suggest that neonatal oxidative stress is linked to an impaired placental oxidative function. Additionally, our analysis showed a positive correlation between umbilical cord protein carbonyl levels and maternal pre-pregnancy weight, BMI, and weight at delivery, indicating that maternal obesity likely contributes to oxidative stress in both the placenta and the newborn. This may play a role in fetal programming, increasing susceptibility to oxidative stress-related diseases later in life [35,50].

Regarding neonatal outcomes, no significant differences were observed in neonatal birth weight or length. However, the growth of the infant was disproportionate to the growth of the placenta, as indicated by a low feto-placental ratio and a high placental weight. Increased placental weight has been associated with greater capillary surface density in cotyledons, enhancing the available surface area for placental exchange and transport functions [51,52]. This enlargement of the placenta demands more nutrients, potentially resulting in inadequate nutrient supply for the developing fetus [53,54]. These findings suggest impaired placental efficiency in the context of metabolic oxidative imbalance, as previously reported [18,54].

However, this study has several limitations. First, it focused exclusively on lean and obese groups; future research should include individuals with overweight pregnancies for a more comprehensive analysis. Second, the study did not perform immunohistochemical assessments of placental vascular density or examine the localization of oxidative stress-related substances within the placenta. Further research in these areas is needed to validate our findings and clarify the underlying mechanisms. Despite these limitations, our study provides evidence that maternal obesity is associated with placental and fetal oxidative stress, along with a concurrent increase in placental antioxidant expression. The neonatal oxidative stress observed in obese pregnancies may contribute to fetal programming, increasing susceptibility to oxidative stress-related diseases later in life. A follow-up study examining the metabolic and oxidative status of children born to mothers with obesity is warranted.

## 4. Materials and Methods

### 4.1. Study Subjects

This study was approved by the Khon Kaen University Ethics Committee for Human Research in adherence with the Declaration of Helsinki and the ICH Good Clinical Practice Guidelines. The study identification code is HE651023. All participants were informed and provided written consent to participate as study volunteers.

A total of 40 pregnant women attending the labor department for delivery at Srinagarind Hospital, Faculty of Medicine, Khon Kaen University, Khon Kaen, Thailand, were recruited for this cross-sectional study. The study groups were classified based on the recommended BMI cut-off points for determining overweight and obesity in Asian populations [2,3] into maternal lean (pre-pregnancy BMI < 23 kg/m^2^) (*n* = 20) and maternal obesity (pre-pregnancy BMI ≥ 25 kg/m^2^) (*n* = 20) groups. Both groups were matched for gestational age at delivery. All volunteers had no history of chronic diseases, including chronic hypertension, diabetes mellitus, and kidney, liver, immune, or respiratory diseases.

### 4.2. Characteristics of the Mothers and Their Offspring

Anthropometric data and pregnancy outcomes, including maternal age, gestational age at delivery, pre-pregnancy weight, pre-pregnancy BMI, weight at delivery, neonatal gender, neonatal birth weight and length, and placental weight, were collected through personal interviews and medical records.

### 4.3. Maternal Plasma Collection

A maternal venous blood sample (10 mL) was collected from the arm veins of the mothers before delivery using ethylenediaminetetraacetic acid (EDTA) tubes. The blood was centrifuged at 3500 rpm for 15 min, and the plasma was separated and stored at −80 °C until analysis. The plasma was used to measure nutritional content (total cholesterol, triglyceride, and protein), oxidative stress markers (MDA and protein carbonyl), and antioxidant activity (SOD and catalase).

### 4.4. Umbilical Cord Plasma Collection

Immediately after the birth of the newborn and the separation of the umbilical cord, the cord was clamped, with another clamp placed 8–10 inches away from the first. A blood sample (10 mL) was promptly collected from the section between the clamps into a tube containing EDTA. The blood was centrifuged at 3500 rpm for 15 min, and the plasma was separated and stored at −80 °C until analysis. The umbilical cord plasma was used to measure nutritional content (total cholesterol, triglyceride, and protein), oxidative stress markers (MDA and protein carbonyl), and antioxidant activity (SOD and catalase).

### 4.5. Placental Sample Collection

Placental samples were randomly harvested within 10 min of delivery. Small fragments of villous tissue (~100 mg) were isolated from the central area of the placenta, placed on ice, and washed with phosphate-buffered solution (PBS) to remove any remaining blood. The samples were then stored at −80 °C until further analysis.

### 4.6. Assessment of Plasma and Placental MDA

The levels of MDA in maternal plasma, umbilical cord plasma, and placental tissue were assessed following a previously described protocol [55]. In brief, 150 μL of plasma was mixed with a stock reagent containing 10% TCA, 5 mmol/L EDTA, 8% SDS, and 0.5 μg/mL BHT. The mixture was incubated at room temperature for 10 min, followed by the addition of 0.6% TBA. The mixture was then boiled in a water bath for 30 min. After cooling, it was centrifuged at 1000× *g* for 10 min. The absorbance of the supernatant was measured at 532 nm using a spectrophotometer. A standard curve was generated using 1,1,3,3-tetraethoxypropane, and the data are expressed as nmol/mg protein.

### 4.7. Assessment of Plasma and Placental Protein Carbonyl

The plasma protein carbonyl content was determined using 2,4-dinitrophenylhydrazine (DNPH) derivatization, followed by spectrophotometric analysis at 370 nm, as previously described [49]. The extinction coefficient of DNPH was used to convert the absorbance into protein carbonyl concentration, expressed as nmol/mg protein.

### 4.8. Assessment of Plasma Catalase Activity

Catalase activity in maternal plasma, umbilical cord plasma, and placental tissue was assessed following a previously described protocol [55]. In brief, 20 µL of plasma or standard solution was added to a microplate, followed by a substrate mixture containing 65 μmol/mL of H₂O₂ in 60 mmol/L PBS (pH 7.4). After incubating at 37 °C for 1 min, 100 µL of ammonium molybdate ((NH_4_)_6_Mo_7_O_24_·4H_2_O) was added to stop the reaction. The yellow complex formed was measured at a wavelength of 405 nm. Catalase from bovine liver (Sigma-Aldrich, Ann Arbor, MI, USA) was used to generate a standard curve, and the data are expressed as U/mg protein.

### 4.9. Assessment of Plasma SOD Activity

Plasma SOD activity was assessed using the SOD Assay Kit-WST (Dojindo Laboratories, Japan) based on colorimetric analysis. The procedure involved adding 20 µL of sample solution to each well, with 20 µL of ultrapure H_2_O added to each blank well. The working solution, dilution buffer, and enzyme solution were then added, followed by incubation at 37 °C for 20 min. The absorbance was measured at 450 nm using a microplate reader. SOD activity was calculated as the % inhibition rate per mg of protein.

### 4.10. Assessment of Plasma Protein Content and Lipids

Maternal blood and umbilical cord blood were used to measure the lipid profiles, including total cholesterol and triglycerides. These measurements were performed at the Community Medical Laboratory, Faculty of Associated Medical Sciences, Khon Kaen University.

Protein concentration in the plasma samples was quantified using the Bradford assay. Briefly, diluted samples were incubated with the Bradford reagent, and the absorbance was measured at 595 nm using a microplate reader. A standard curve, generated using a bovine serum albumin (BSA) protein standard, was used to convert the absorbance values into protein concentrations.

### 4.11. Placental Protein Extraction and Expression Analysis

The placental samples were homogenized in PBS containing proteolytic enzyme inhibitors (Complete-Mini; Roche, Basel, Switzerland) using an Ultra-Turrax homogenizer (Bioblock Scientific, Illkirch, France). After homogenization, the samples were centrifuged at 4000 rpm for 30 min, and the supernatant (protein extracts) was collected for Western blot analysis.

Placental antioxidant protein expression was measured using 30–50 µg of protein per sample. The proteins were separated on 10–12% SDS-PAGE gels. Primary antibodies against SOD2 (Santa Cruz Biotechnology, Heidelberg, Germany; 1:1000), catalase (Sigma-Aldrich, St. Louis, MO, USA; 1:2000), UCP2 (Santa Cruz Biotechnology, Heidelberg, Germany; 1:1000), and Nrf2 (Santa Cruz Biotechnology, Heidelberg, Germany; 1:500) were applied overnight at 4 °C. After washing, a secondary antibody (anti-mouse IgG peroxidase-conjugated) was applied for 1 h at room temperature. The blots were washed again and incubated with enhanced chemiluminescence reagents (ECL Prime, Amersham Bioscience, Little Chalfont, Buckinghamshire, UK), and the bands were detected using the ChemiDoc XRS+ imaging system (Bio-Rad, Ann Arbor, MI, USA). The blots were re-incubated with a β-actin antibody (Santa Cruz Biotechnology, Heidelberg, Germany; 1:4000) as an internal control. The expression levels of SOD2, catalase, UCP2, and Nrf2 were normalized with β-actin.

### 4.12. Statistical Analysis

The data were statistically analyzed using GraphPad Prism software version 9.3.1 (GraphPad, East Lansing, MI, USA). The Kolmogorov–Smirnov test was used to assess data normality. Normally distributed data are expressed as mean ± standard error of the mean (SEM). Comparisons between the two groups were performed using Student’s *t*-test and Pearson’s Chi-square test. Correlations between parameters were evaluated using Pearson’s correlation test. Differences were considered statistically significant when *p* < 0.05.

## 5. Conclusions

This study provides evidence that maternal obesity disrupts the maternal–fetal–placental oxidative balance, with neonatal oxidative stress linked to impaired placental oxidative function. The oxidative stress observed in neonates from obese pregnancies may be a key factor in fetal programming, increasing the susceptibility to oxidative stress-related diseases later in life. Understanding the relationship between neonatal and placental oxidative stress could offer valuable insights into oxidative damage caused by maternal obesity and help inform strategies to mitigate this damage.

## Figures and Tables

**Table 1:** Demographics and pregnancy outcomes.

Parameter	Maternal Lean	Maternal Obesity	*p*-Value
Maternal age at delivery (year)	27.65 ± 0.85	30.30 ± 1.20	0.0779
Gestational age at delivery (weeks)	38.71 ± 0.21	38.81 ± 0.28	0.7744
Pre-pregnancy weight (kg)	52.56 ± 1.29	75.32 ± 1.79 ***	<0.0001
Pre-pregnancy BMI (kg/m^2^)	20.26 ± 0.45	29.40 ± 0.58 ***	<0.0001
Weight at delivery (kg)	66.90 ± 2.10	90.64 ± 2.22 ***	<0.0001
Vaginal delivery *n* (%)/ Cesarean delivery *n* (%)	13 (65)/7 (35)	8 (40)/12 (60)	0.1134
Neonatal sex, male *n* (%)	10 (50)	11 (55)	0.7575
Neonatal birth weight (g)	3083 ± 101	3327 ± 110	0.1103
Neonatal length (cm)	49.20 ± 0.57	49.21 ± 0.59	0.9898
Placental weight (g)	581.50 ± 28.02	701.50 ± 41.89 *	0.0224
Feto-placental ratio	5.43 ± 0.19	4.90 ± 0.16 *	0.0374

Results are reported as mean ± SEM or *n* (%). *n* = 20 for each group. *p*-value was tested using Student’s *t*-test for continuous variables and Pearson’s Chi-square test for categorical data. * *p* < 0.05; *** *p* < 0.0001 relative to the maternal lean group.

**Table 2:** Correlation between umbilical cord protein carbonyl level and placental oxidative status, as well as with maternal oxidative status and anthropometric parameters.

Umbilical Cord Protein Carbonyl	r	*p*-Value
Placental protein carbonyl	0.7405	<0.0001 ***
Placental catalase expression	−0.3942	0.0566
Placental SOD2 expression	−0.1674	0.4343
Placental UCP2 expression	0.0654	0.8099
Placental Nrf2 expression	−0.3546	0.0891
Maternal protein carbonyl	−0.09101	0.5765
Maternal catalase activity	−0.4332	0.0052 **
Maternal SOD activity	−0.2246	0.1635
Maternal pre-pregnancy weight	0.3621	0.0217 *
Maternal pre-pregnancy BMI	0.3832	0.0147 *
Maternal weight at delivery	0.3168	0.0464 *

The correlation of the parameters was assessed by using Pearson’s correlation test; * *p* < 0.05; ** *p* < 0.01; *** *p* < 0.001.

## Data Availability

The data are contained within the article. Additional raw data can be obtained from the corresponding author on request.
